# Sample representativeness and temporal comparability in SEAR study

**DOI:** 10.1016/j.lansea.2025.100646

**Published:** 2025-07-29

**Authors:** Jabed Iqbal

**Affiliations:** SingHealth Duke-NUS Global Health Institute, 8 College Road, 169857, Singapore

I commend Singh and colleagues^1^ for their multilevel analysis of health insurance coverage in six WHO Southeast Asia Region (SEAR) countries using Demographic and Health Surveys (DHS) data. Their gender-disaggregated findings, showing coverage ranging from 0% in Bangladesh to 94.8% in the Maldives among women, advance the discourse on Universal Health Coverage (UHC). In this Correspondence, I raise two methodological points to enhance generalisability.

First, India's dominance (86.3% of female, 80% of male respondents [Table 2 in the research article^1^]) may bias pooled regional estimates and community-level heterogeneity. Sensitivity analyses excluding India or weighting by country population could improve regional applicability. Second, the wide span of the 2015–2022 DHS data risks comparability due to reforms like India's *Ayushman Bharat* (2018) and Indonesia's *Jaminan Kesehatan Nasional* expansion.^2^ Aggregating datasets may mask policy impacts. Stratifying by survey year or using a shorter timeframe (e.g., 2019–2022) could ensure valid comparisons.

Notably, women in the richest quintile had lower odds of insurance coverage than those in the poorest quintile, suggesting substitution effects or confounders (Table 4 in the research article^1^). This counterintuitive finding merits further study, particularly regarding private versus social security insurance dynamics. Overall, despite challenges in multi-country analyses due to shifting policies, the study retains its value.

Additionally, the Maldives is notable in the SEAR for its health insurance coverage, which is supported by a universal, government-funded system and equity-focused policies, and serves as a reference for other SEAR countries aiming to improve insurance and healthcare access. Exploring how relatively high-performing countries like the Maldives address gender disparities in healthcare could further enrich the study's insights, contributing to health equity and UHC in SEAR.

## Contributors

JI conceptualised, drafted, and edited this manuscript.

## Declaration of interests

I declare no competing interests.
